# Memory load differentially influences younger and older users’ learning curve of touchscreen gestures

**DOI:** 10.1038/s41598-022-15092-y

**Published:** 2022-06-25

**Authors:** Bingxin Li, Tong Yang, Yanfang Liu, Feng Du

**Affiliations:** 1 CAS Key Laboratory of Behavioral Science, Institute of Psychology, Chinese Academy of Sciences, Beijing, China; 2 Department of Psychology, University of Chinese Academy of Sciences, Beijing, China; 3 Huawei & Chinese Academy of Sciences UX Design Human Factors Joint Lab, Beijing, China

**Keywords:** Human behaviour, Cognitive ageing, Learning and memory

## Abstract

In this study, we employed a recall test to investigate how memory load affects the learning curve of gesture-letter pairs for younger and older users. The gesture-letter pairs were carefully designed to mimic real-world gesture-function/command associations on a touchscreen mobile phone. Both younger and older user groups showed lower recall accuracy as the memory load of gesture-letter pairs increased, and recall performance improved with repeated memory training. More specifically, younger users improved rapidly over repeated memory sessions under all memory loads, whereas older users benefited much less from repeated memory sessions except the lowest memory load of 6 gesture-letter pairs. These results reveal that the memory load differentially modulated younger and older users’ learning curves of gesture-letter pairs. Thus, our work suggests an upper limit when adding new gesture-function associations on mobile phones and special attention should be devoted to old users.

## Introduction

Touchscreen mobile phones have become a pivotal device for people’s daily life because of its powerful and flexible connection with various function tools, with engaging users from a young age until the old. It has been shown that in 2020 the average smartphone user had 40 applications installed on the phone^1^. Users usually spend hours a day on their phones, and often find it difficult to execute core system commands (e.g., phone language setting, mobile data/WiFi/Bluetooth on/off) and access various applications (e.g., starting a timer, browsing a website, and opening a favorite contact). They need to navigate through a deep interface hierarchy, which is time-consuming. Thus designers hope to provide as many navigational gestures as possible to help users perform tasks. There are already some navigational gestures available on the existing operating systems. For example, users can return to home page by swiping up quickly from the bottom of the screen on Android or iOS systems. Similarly, by holding a finger on the screen after swiping up from the bottom edge in both systems, users can open the recent application view displaying swipeable cards of all running applications.

Android system has introduced many more complex navigational gestures than iOS. For example, knocking firmly with knuckle while sliding horizontally across the screen will enable split-screen mode. Moreover, a double knock or drawing a letter “S” with a knuckle anywhere on the screen can take a screenshot. However, new users and elderly users may find it difficult to memorize so many complex gestures. Users may not want to have a heavy burden of gesture learning. However, it is unknown that how many pairs of gestures and the related action can be memorized and how efficiently users of different ages can learn those pairs with repeated practice.

There are a few empirical studies that evaluated the memorability of touchscreen gestures and commands using recall tests^2–4^. For example, Nacenta et al.^2^ found that user-defined gestures are easier to be memorized by users when compared with pre-designed gesture set and random set. Researchers argued that user-defined gestures may be more memorable than system-defined gestures, because users have established a strong association between gestures and actions when defining the gestures. On average the recall rate of self-defined gesture shortcuts was above 90%^3^. In addition, user-defined gestures were more memorable compared to system-defined letter gestures (i.e., draw a letter to evoke function)^3^. Indeed, associating a specific command with semantic gestures such as a letter or multiple letters can be a challenging task, especially for users who use symbolic language. Thus, instead of studying letter gestures, in the present work we employed unistroke swipes (a single swiping movement) which mimicked the existing set of fullscreen navigational gestures. Moreover, every swipe gesture was randomly assigned with a unique letter representing a command/action to be executed (rather than applying letter gestures) in order to understand the memorability of pair between gestures and the associated actions as the pair number increases. According to previous results of the list length effect on memory performance^5–7^, the mean proportion of items recalled decreases as the length of the memory list increases. Supporting evidence also comes from change-detection tasks, in which detection accuracy also declined when memory load increased^8,9^. Thus, we predicted lower memory accuracy for gesture-letter pairs as the memory load became higher.

It is well-known that repeated learning and testing can significantly improve recall performance^10–15^. For example, a study showed that, compared to one presentation, five-fold repetition of word pairs increased both the familiarity of every individual word in the pair and the associative strength of the word pairs, leading to higher memory accuracy^13^. In addition, repeated learning and testing of the same words contributed to a higher proportion of recall of words and its category^14^. Thus, in two experiments by applying five sessions of repeated learning and testing method, we predicted that users would on average show increased accuracy in recalling pairs of gestures and the associated letter, but the learning rate of gesture-letter pairs would be slower as the memory load increased.

Furthermore, in the second experiment we planned to examine how fast younger and older users would learn the gesture-letter pairs under different memory loads. Aging and memory have been elaborated in the past research^16–22^. Studies consistently show a significant age decline in episodic memory. Performance on memory tests requiring association is particularly vulnerable to aging^18,20–22^. In a classic study on aging and memory for relationship among items, researchers found that older adults performed poorly in recognition of various types of associative information (e.g., word-nonword pairs, word-font pairs), suggesting greater difficulty in encoding and retrieving specific associations between units of information in the aged group^21^. With a rather different context using a gesture learning test, we predicted better recall performance in the younger than in older users, and their differences would be larger under high memory load.

In sum, the present study contains reports of two experiments. Experiment 1 aimed to examine the learning curves for two different memory loads of gesture-letter pairs. Some of the gesture-letter pairs in the first experiment were designed to imitate the existing navigational gestures and the command to be executed. Experiment 2 was conducted, by employing a wider range of levels of memory loads, to investigate the memory performance in users of different ages under different memory loads and after repeated practice. The present study may provide important information for UI designers on how many gestures are easy for users of different ages to memorize.

## Experiment 1

In the present experiment, we asked users to recall the letter associated with each gesture (unistroke swipe gestures) after every memorizing session for five times. We planned to examine the learning curve (memory performance across memorizing sessions). Subjective data including perceived learning effectiveness, general evaluations, fatigue and emotional experience for two different memory loads of gesture-letter pairs were also assessed.

### Experiment 1 methods

#### Users

The study was approved by the Ethics Committee of Human Experimentation at the Institute of Psychology, Chinese Academy of Sciences. All procedures were performed in accordance with relevant guidelines and regulations.

Sample size was estimated based on prior studies on memory load/list length (*η*^*2*^_*p*_ > 0.75)^5,6^ and repetition/testing effect on recall performance (*η*^*2*^_*p*_ > 0.30)^13^. A statistical power analysis using G*power^23^ indicated an optimal sample size of *N* = 9 if the effect size *η*^*2*^_*p*_ = 0.30 wi*th α* = 0.05, and power = 0.90.

Eighteen users who had experience of touchscreen mobile phones (10 females; mean age = 26.89 years, *SD* = 10.47, age ranged between 21 and 38 years) took the experiment in exchange for monetary compensation (see Appendix A for demographic details). All users reported having normal or corrected-normal vision. They signed an informed consent form before the experiment.

#### Apparatus, materials and procedure

**Figure 1:**
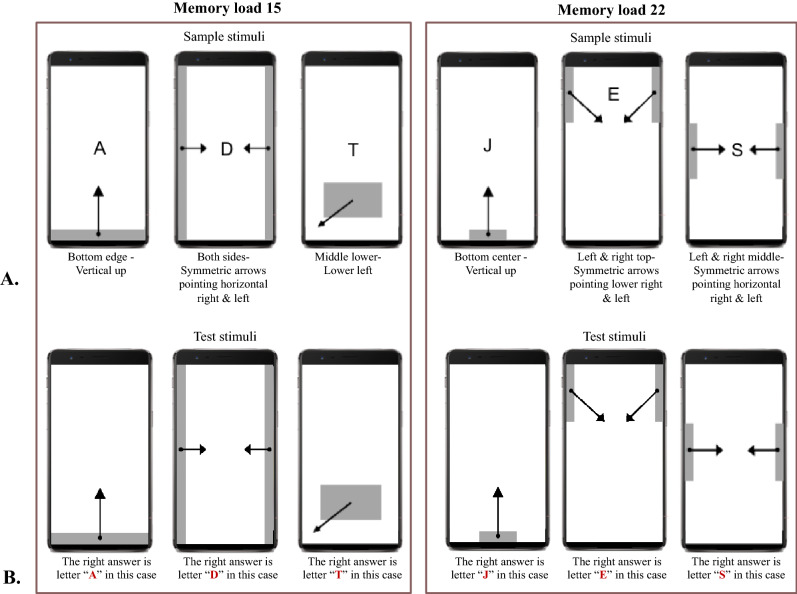
Examples of gesture-letter pairs in each memory load (**A**) and the related test stimuli (**B**).

As Fig. 1 showed, pictures of swiping gestures (pointing vertically, horizontally or in diagonal directions from screen edges or middle-lower part) were presented on a 14-inch ThinkPad laptop. Every gesture appeared in pair with a unique letter presented at the center of the screen (see examples in Fig. 1 and all gesture-letter pairs in Appendix B). The gesture-letter pairs were prespecified by E-prime 2.0.10 (Psychology Software Tools, Pittsburgh, PA, USA), showing in a pseudorandom order.

There are two memory loads of gesture-letter pairs: 15 and 22 pairs (see Appendix B). For the memory load 15 condition, users needed to learn gestures swiping from the bottom edge, middle lower and left/right sides of the screen and the associated letters. In this condition, some of the gestures and the associated letter were designed to imitate the existing fullscreen navigational gestures and the related action, e.g., back to home screen by swiping up from the very bottom of the screen. For the memory load 22 condition, we required users to swipe from a narrower starting region in order to have more complex gesture-letter pairs. In this condition, a limited number of directions were included if swiping from an area that was close to the four corners of the screen; and in this condition some of the gesture-letter pairs imitated the existing complex navigational gestures, e.g., the corner swipe gesture to invoke Google assistant on Android. In both memory load conditions, symmetric gesture directions on the left and right sides of the screen were always shown simultaneously, assigned with the same letter.

In order to make sure that users understood the task, they were given a few pairs of swiping gestures and digits for practice before the real experiment. Note that gesture-digit pairs were only used in the practice and did not appear in the experiment to avoid confusion. In the critical experiment, users were given five memorizing sessions repeatedly, each of which was followed by a recall test of gesture-letters pairs—the standard condition in free recall learning^24^. In every memorizing session, gesture-letter pairs were presented in random order. Each pair appeared on screen for 5 s, then a 500 ms blank interval before the onset of the next pair. In the recall test users had to retrieve the corresponding letter for a specific gesture just learned, without feedback. Users had as much time as they needed to provide a response. After five sessions of gesture memorizing and testing of every memory load condition, a survey of easiness to learn and memorize, general evaluation of gestures, perceived fatigue and emotional experience was applied (Appendix C). Perceived fatigue and emotional experience were assessed because it has been suggested that fatigue and emotional response are important aspects for evaluating user experience^25,26^. For all users memory load 15 was performed before 22 so that they would not be too frustrated at the beginning of the study. Breaks were allowed after every recall test. The whole experiment lasted about 60 min (including break time).

#### Design

The memory load (15 or 22 gesture-letter pairs) and memorizing session (S1, S2, S3, S4, S5) were two within-subject variables. The recall accuracy, fatigue and emotions rating, perceived easiness of learning and memorizing as well as general evaluations of gestures were key dependent variables.

### Experiment 1 results

#### Recall accuracy

Recall accuracy was analysed using a repeated measures ANOVA with memory load and memorizing session as within-subject factors, though the data were nonnormally distributed for three reasons. First, most studies on recall memory used ANOVA to assess memory loads’ effect (also the effects of repeated learning^13,14^ or age groups^15–18^) on recall accuracy. Secondly, Norman^27^ had shown that ANOVA can also be used for data with non-normal distributions and data of Likert scale. Lastly, only a few non-parametric tests (e.g., Scheirer–Ray–Hare test, aligned ranks transformation ANOVA) can be used for two-way factorial design. However, they are not suited for detecting an interaction.

**Figure 2:**
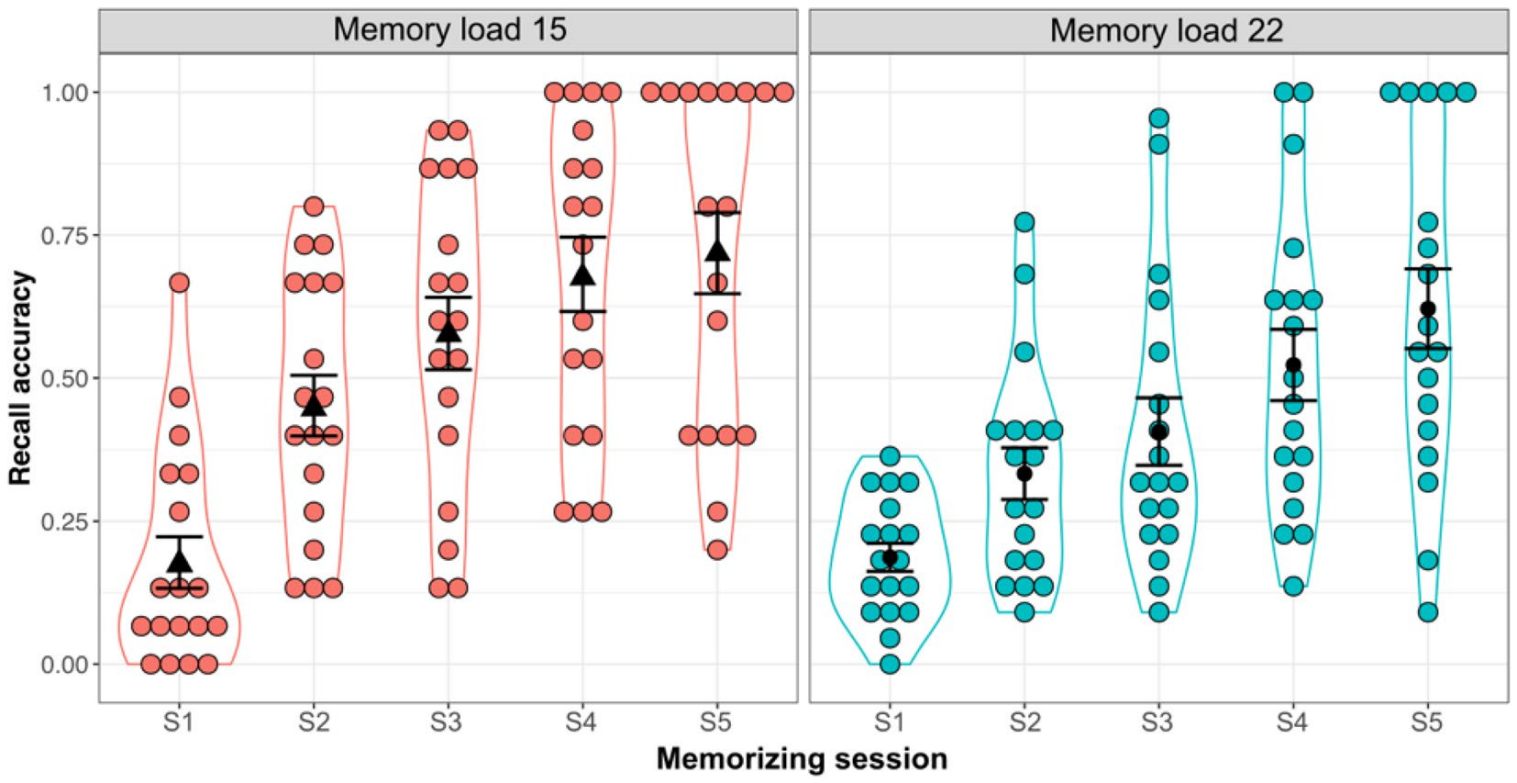
Mean recall accuracy as a function of memory load and memorizing session. Black triangles and dots represent mean recall accuracy in the memory load 15 and 22, receptively, following every memorizing session. Jittered dots in red are recall accuracy for each user in the memory load 15 and the dots in green are individual recall accuracy in the memory load 22. Error bars indicate the standard error of the mean.

Accuracy for each condition is illustrated in Fig. 2 and shown in Appendix D. There was a significant main effect of memory load, *F*(1, 17) = 5.75, *p* = 0.028, *η*^*2*^_*p*_ = 0.25, with higher recall accuracy in the memory load 15 (mean accuracy of 0.52) compared to the memory load 22 (0.41). The effect of memorizing session was significant, *F*(4, 68) = 49.84, *p* < 0.001, *η*^*2*^_*p*_ = 0.75, with higher accuracy with more memory training. In addition, memory load interacted significantly with memorizing session, *F*(4, 68) = 3.23, *p* = 0.017, *η*^*2*^_*p*_ = 0.16. Post hoc analysis revealed that the improvement in the memory load 15 (accuracy in S5—accuracy in S1 =  + 0.54, *p* < 0.001) appeared to be slightly larger than in the memory load 22 (accuracy in S5—accuracy in S1 =  + 0.43, *p* < 0.001). In particular, recall accuracy of first memorizing session in the memory load 15 was not different from that of memory load 22, *t*(26.31) = − 0.18, *p* = 0.861, Cohen’s *d* = 0.06.

#### Gesture evaluation scores

**Figure 3:**
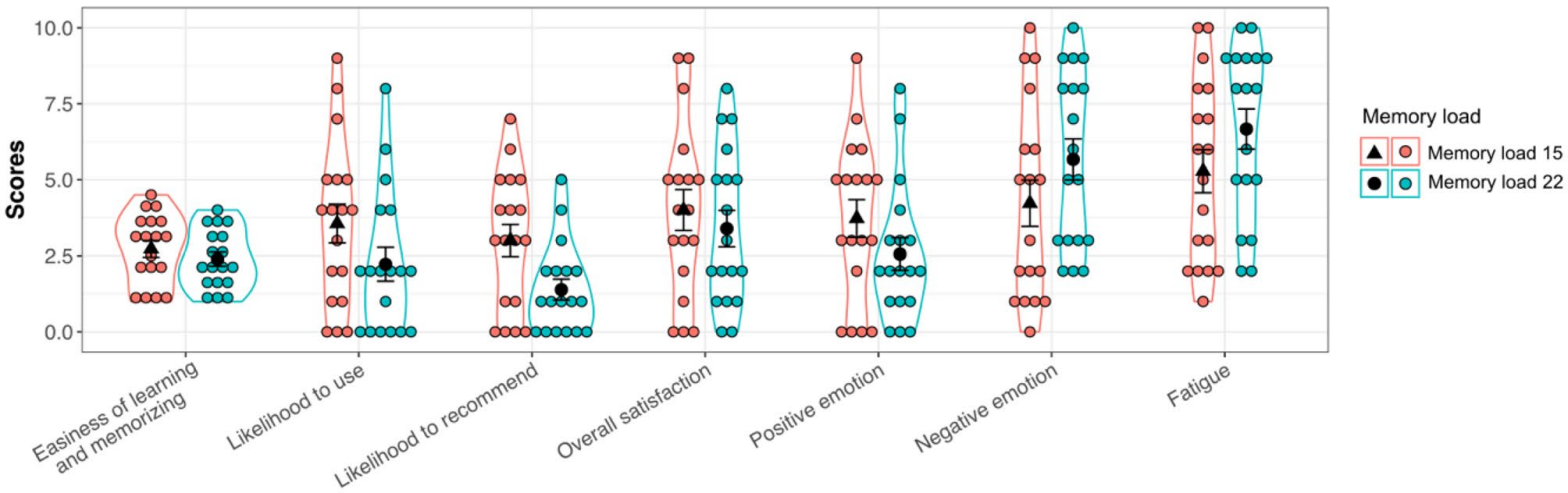
Mean subjective rating scores of gestures. Black triangles and dots represent mean scores of subjective ratings in the memory load 15 and memory load 22, receptively. Jittered dots in red and those in green are rating scores for each user in the memory load 15 and memory load 22, respectively. Error bars indicate the standard error of the mean.

Seven subjective rating scores for two sets of gesture-letter pairs (e.g., ease of learning, likelihood to use et al.) were compared using paired sample t-tests. Mean rating scores and the corresponding SEs for different memory loads are illustrated in Fig. 3 and shown in Appendix E.

Perceived easiness of learning and memorizing in the memory load 15 was not different from that of memory load 22, *t*(17) = 1.60, *p* = 0.128, Cohen’s *d* = 0.31; neither was overall satisfaction, *t*(17) = 1.05, *p* = 0.310, Cohen’s *d* = 0.23. However, users indicated higher likelihood to use the memory load of 15 gesture-letter pairs than the memory load 22, *t*(17) = 2.82, *p* = 0.012, Cohen’s *d* = 0.53; they were also more likely to recommend the 15 pairs of gesture set than 22 pairs, *t*(17) = 1.61, *p* = 0.003, Cohen’s *d* = 0.86.

#### Fatigue and emotional experience

Users also reported significantly higher positive emotion in the memory load 15 compared to 22, *t*(17) = 2.93, *p* = 0.009, Cohen’s *d* = 0.48; lower rating of negative emotion, *t*(17) = − 2.15, *p* = 0.046, Cohen’s *d* = 0.47; and lower levels of fatigue, *t*(17) = − 3.65, *p* = 0.002, Cohen’s *d* = 0.48.

### Summary of experiment 1

Experiment 1 showed lower recall accuracy in memory load of 22 pairs (mean accuracy of 41%) than in memory load of 15 pairs (52%). Moreover, recall performance significantly improved by repeated learning and testing of the same set of gesture-letter pairs. Especially there were larger and faster improvements for the memory load of 15 pairs than the memory load of 22 pairs, revealing users can learn associative binding of gestures and letters more rapidly under low memory load.

Users were also more likely to use and to recommend the memory load 15 compared to 22, with better emotional experience and lower level of fatigue for the memory load 15. However, users reported no significant differences in the easiness of learning and memorizing and ratings of overall satisfaction between the two memory loads.

## Experiment 2

Experiment 2 aimed to examine whether memory load differentially influences the younger and older users’ gesture learning curve over memorizing sessions. Different from Experiment 1 where memory loads of 15 and 22 gesture-letter pairs were tested, in this experiment we applied a wider range of memory load levels varying between 6 and 22 gesture-letter pairs to investigate the upper limit of gesture learning in the users of different ages.

### Experiment 2 methods

#### Users

The sample size of each age group was estimated based on prior studies on memory performance and aging (Cohen’s *d* > 1.39)^16,18,21^. A statistical power analysis^23^ indicated an optimal sample size of *N* = 12 in each group if the effect size Cohen’s *d* = 1.39 with *α* = 0.05, and power = 0.90.

Twelve younger smartphone users (7 females; mean age = 27.00 years, *SD* = 5.38, age ranged between 19 and 38 years) and 12 older smartphone users (6 females; mean age = 54.83 years; *SD* = 4.71, age ranged between 45 and 63 years) who had normal or corrected-to-normal vision took part in the experiment in exchange for monetary compensation. All users provided written informed consent before the experiment. The two age groups had similar years of experience in touchscreen smartphone use (*χ*^2^(3, 24) = 2.00, *p* = 0.572; see Appendix A for demographic details).

#### Design

The experiment was a three-factor (2 × 4 × 5) mixed design. User group (younger, older) served as a between-subject factor whereas memory load (6, 9, 18 or 22 gesture-letter pairs) and memorizing session (S1, S2, S3, S4, S5) were two within-subject factors. The recall accuracy, fatigue and emotions rating, perceived easiness of learning and memorizing as well as general evaluations of gestures were used as key dependent variables as in Experiment 1.

#### Apparatus, materials and procedure

The apparatus was identical to those used in Experiment 1. Different from Experiment 1, gesture-letter pairs were shuffled matching different letters with swipe gestures, additionally, memory load levels were manipulated varying between 6 and 22 gesture-letter pairs (Appendix F). Specifically, memory load 6 and 9 were both simplified conditions of the memory load 15 in Experiment 1, and memory load 18 was a simplified condition of memory load 22 in Experiment 1. The present experiment also included memory load 22 which was different from the same condition in Experiment 1 but had gestures swiping from a much narrower start area on the screen edges. Identical to Experiment 1, in each memory load condition, symmetric gesture directions on the left and right sides of the screen were always shown simultaneously, assigned with the same letter. Similar to Experiment 1, users were given instructions and practice trials (a few gestures-digit pairs) to familiarize themselves with the task. All users completed the task in the following order: memory load 6, memory load 9, memory load 18 and memory load 22 to avoid frustration at the beginning of the study. Breaks were allowed after every recall test. The whole experiment lasted about 90 min (including break time).

### Experiment 2 results

#### Recall accuracy

**Figure 4:**
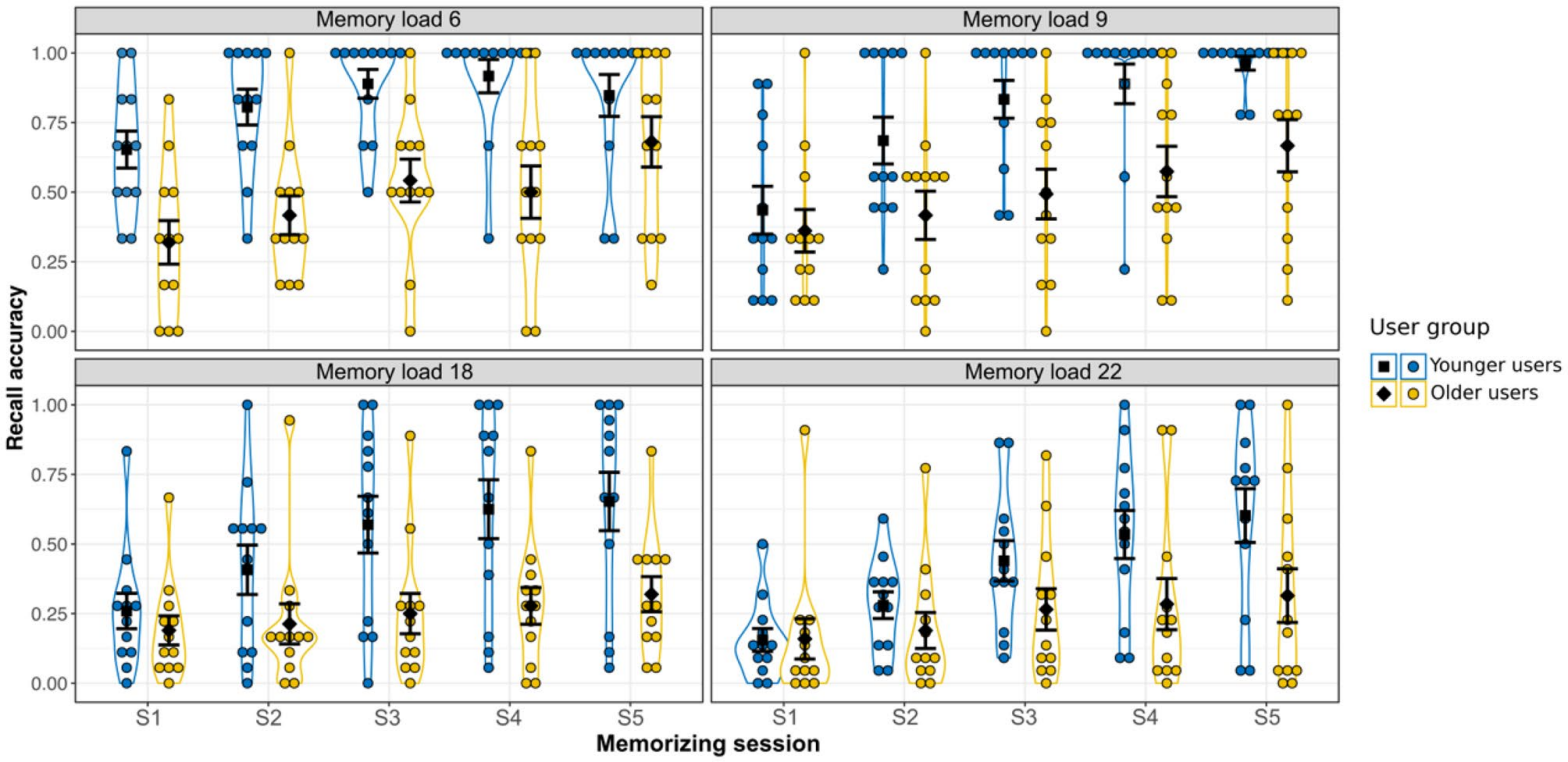
Mean recall accuracy as a function of memory load, memorizing session and user group. Black squares and diamonds represent mean recall accuracy in the younger and older users, receptively, for the different memory loads following every memorizing session. Jittered dots in blue are recall accuracy for every younger user and the dots in yellow are individual recall accuracy of older users. Error bars indicate the standard error of the mean.

Recall accuracy was analyzed using a mixed measures ANOVA with memory load and memorizing session as within-subject factors and user group as a between-subjects factor, though the data were nonnormally distributed1^13–18,27^. Accuracy for each condition is illustrated in Fig. 4 and shown in Appendix G. There was a significant main effect of memory load, *F*(3, 66) = 27.70, *p* < 0.001, *η*^*2*^_*p*_ = 0.56. Post hoc analysis showed significantly lower recall accuracy in the memory load of 18 and 22 pairs compared to the memory load of 6 (all *p* < 0.001) and 9 pairs (all *p* < 0.001). Recall accuracy was even lower in the memory load 22 compared to 18 (*p* = 0.007). Recall accuracy in memory load 6 was not significantly different from memory load 9 (*p* = 0.402). The effect of memorizing session was significant, *F*(4, 88) = 41.59, *p* < 0.001, *η*^*2*^_*p*_ = 0.65. Post hoc test indicated improved recall accuracy with more sessions of memory training (accuracy in S5 – accuracy in S1 =  + 0.31, *p* < 0.001). The main effect of user group was also significant, *F*(1, 22) = 11.16, *p* = 0.003, *η*^*2*^_*p*_ = 0.34. Younger users (mean accuracy of 0.62) recalled more gesture-letter pairs indicating a higher level of accuracy compared to older users (0.37).

Importantly, user group interacted significantly with memory load and memorizing session to predict recall accuracy, *F*(12, 264) = 2.17, *p* = 0.014, *η*^*2*^_*p*_ = 0.09. By performing four additional 2 (user group) × 5 (memorizing session) ANOVAs with mixed measurements we investigated whether there were differences between the younger and older users in their learning curve for each memory load. Results are detailed in Appendix H.

To sum up, the main effect of memorizing session was significant for all memory loads. Users had higher recall accuracy with more sessions of memory training. However, we found a significant interaction between memorizing session and user group for all memory loads except the memory load 6. Post hoc comparisons indicated that for the memory loads of 9, 18 and 22 pairs younger users showed significant improvement over memorizing sessions, whereas the effect was not reliable in the older users. For memory load 6 both groups had increased accuracy with more sessions.

#### Younger and older users’ recall accuracy of the first and last memorizing session

The two-sample independent t-test was performed in each memory load condition to check whether the two user groups show significant differences in recall accuracy during the first memorizing session. We found that younger users outperformed older users in the memory load 6, *t*(21.45) = − 3.26, *p* = 0.003, Cohen’s *d* = 1.33. However, recall accuracy was similar for younger and older users in the memory load 9, *t*(21.70) = − 0.64, *p* = 0.526, Cohen’s *d* = 0.26; memory load 18, *t*(21.21) = -0.84, *p* = 0.408, Cohen’s *d* = 0.34; and memory load 22, *t*(17.37) = 0.05, *p* = 0.964, Cohen’s *d* = 0.02.

In the last memorizing session, similar analyses were performed. We found that recall accuracy was significantly higher in the younger than older users for memory load 9, *t*(12.55) = − 3.05, *p* = 0.010, Cohen’s *d* = 1.24; memory load 18, *t*(18.07) = − 2.73, *p* = 0.014, Cohen’s *d* = 1.11; and memory load 22, *t*(22) = − 2.11 *p* = 0.046, Cohen’s *d* = 0.86. For memory load 6 both groups showed comparable accuracy, *t*(21.29) = − 1.42, *p* = 0.171, Cohen’s *d* = 0.57.

#### Gesture evaluation scores

**Figure 5:**
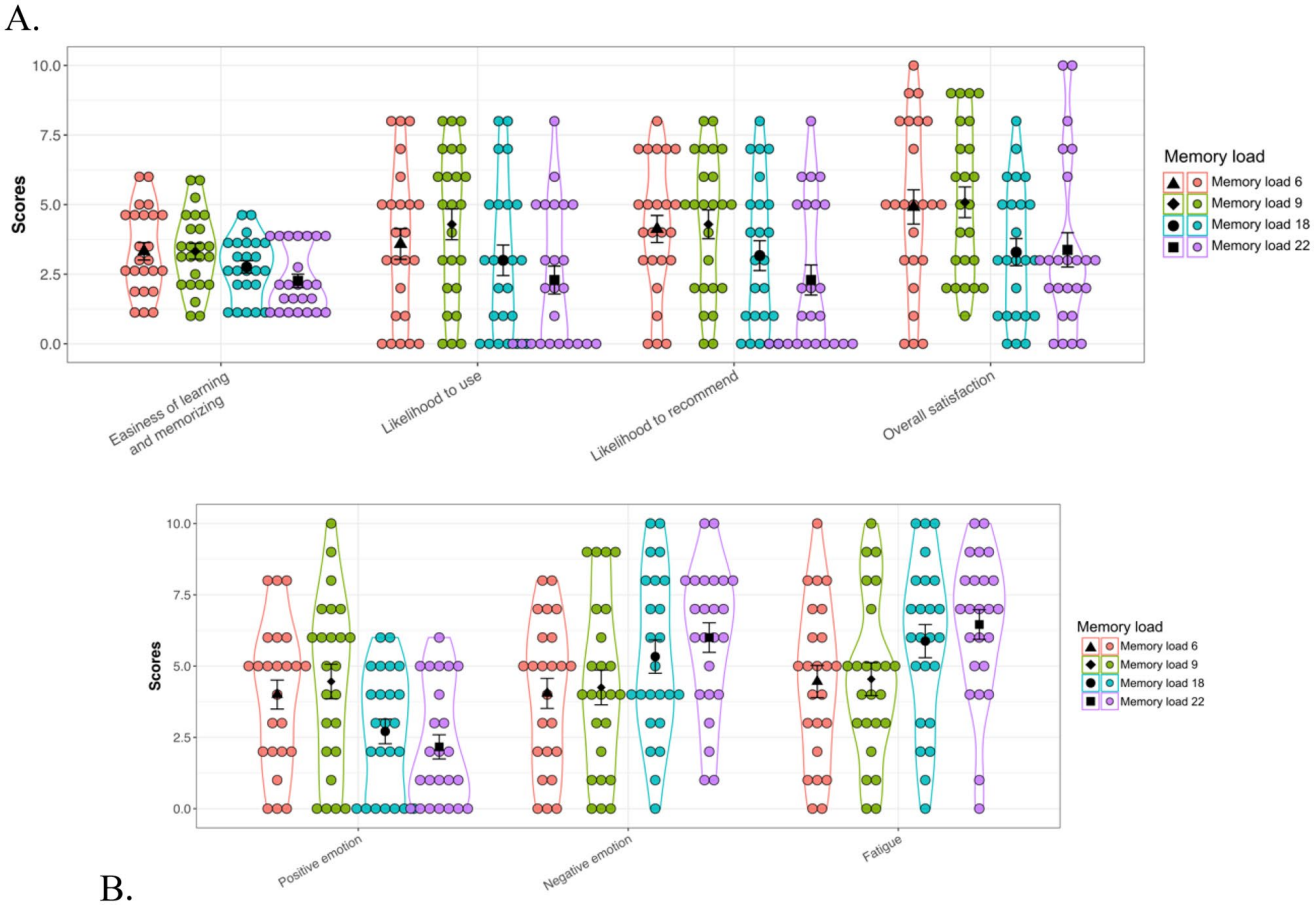
Mean rating scores of gesture evaluations (**A**) and emotions and fatigue (**B**). Black triangles, diamonds, dots and squares represent mean scores of subjective ratings in the memory load 6, 9, 18 and 22, receptively. Jittered dots in red, green, blue and purple are individual rating scores in the memory load 6, 9, 18 and 22, respectively. Error bars indicate the standard error of the mean.

Subjective rating scores for four memory loads of gesture-letter pairs were compared between the two user groups using two-way ANOVA with mixed measurements. Mean rating scores and the corresponding SEs for every memory load are illustrated in Fig. 5 and shown in Appendix I.

The main effect of user group was not significant for all gesture evaluation scores (all *p* > 0.05). However, we found a significant main effect of memory load. Specifically, perceived easiness of learning and memorizing, *F*(3, 66) = 6.54, *p* < 0.001, *η*^*2*^_*p*_ = 0.22; likelihood to use, *F*(3, 66) = 7.27, *p* < 0.001, *η*^*2*^_*p*_ = 0.25, likelihood to recommend, *F*(3, 66) = 10.58 *p* < 0.001, *η*^*2*^_*p*_ = 0.32, and overall satisfaction, *F*(3, 66) = 6.47, *p* < 0.001, *η*^*2*^_*p*_ = 0.23, were significantly different across the four memory load conditions. Post hoc tests showed that, in general, gesture evaluation scores associated with the memory load 6 and 9 were relatively high, whereas the score decreased as increases in memory load. The two-way interaction between user group and memory load did not reach significance on any gesture evaluation score (all *p* > 0.05).

#### Fatigue and emotional experience

Again, by applying a two-way ANOVA we found that the main effect of user group was not significant for perceived fatigue and emotional experience (all *p* > 0.05). However, we found a significant main effect of memory load on perceived fatigue, *F*(3, 66) = 11.34, *p* < 0.001, *η*^*2*^_*p*_ = 0.34; positive emotion, *F*(3, 66) = 10.13, *p* < 0.001, *η*^*2*^_*p*_ = 0.32; and negative emotion, *F*(3, 66) = 6.34, *p* < 0.001, *η*^*2*^_*p*_ = 0.22. Post hoc analyses revealed that memory load of 6 and 9 pairs were associated with lower scores on fatigue and negative emotion, but higher scores on positive emotion, compared to memory load of 18 and 22 pairs. The two-way interaction did not reach significance on fatigue and emotional experience (all *p* > 0.05).

### Summary of experiment 2

Experiment 2 replicated the results of Experiment 1 that users on average showed reduced recall accuracy and lower gesture evaluation scores when memory load was high. Importantly, we found that younger users were higher in memory accuracy compared to older users. Younger users on average had an accuracy of over 85% in the memory load of 6 and 9 gesture-letter pairs, and they even reached 60% for the higher memory loads (18 and 22 pairs) by the end of the experiment (S5). In contrast, worse performance was found for older users who had an accuracy of below 70% when tested with the memory load of 6 and 9 pairs. Their accuracy was just around 30% or lower in the memory load 18 and 22.

We observed increases in recall accuracy with more sessions of memory training as in Experiment 1. Moreover, the present experiment identified a larger improvement in younger than older users over memorizing sessions. For every memory load condition, five sessions of memory training helped younger users to form a stronger association between gestures and letters, but the training was less helpful for older users. This was not the case, however, for the memory load of 6 gesture-letter pairs, where both groups of users indicated significant increases in recall accuracy over sessions, as a result, they even showed matched performance in the last session. Subjective measures were not different between the two groups for any memory load conditions.

## General discussion

In the two experiments, as predicted, we found lower recall accuracy when the memory load increased for both older and younger users, which is consistent with previous findings of poorer memory performance associated with a longer study list^5,6^. Studies proposed that differences in memory performance between short and long lists can be accounted for by more attention paid to the stimulus of the short list while comparatively less attention to each stimulus of the long list^28^. In line with our prediction, we also showed that memory performance of gesture-letter pairs can be significantly improved by five successive memorizing sessions, the so-called “learning effect” of repeated learning and testing^10^. The learning effect was identified in previous studies on free recall^11–13^ and paired-associates recognition^14,15^.

Relative to younger users, older users performed more poorly in the recall test of pairs of gestures and the related letter. Importantly, we found that the learning curve of gesture-letter pairs was different between the two user groups. In the first memorizing session of the experiment, both younger and older users had similar levels of recall accuracy for the memory load 9, 18 and 22. However, we only observed a significant improvement in younger users’ recall accuracy but not in older users, indicating learning deficits in aged users compared with younger users^16–22^. In contrast, for memory load 6, though older users recalled fewer gestures-letter pairs compared to younger users in the first memorizing session, both groups significantly improved their recall accuracy over memorizing sessions. Consequently, younger and older users had comparable recall accuracy after five sessions of memory training. These results are consistent with our last hypothesis predicting better recall performance in the younger than in older users under high memory load conditions.

Differences in the learning curve of gestures between younger and older users are most likely due to age-related deficits in the associative binding process. Previous studies suggested that declines of episodic memory with aging are mainly due to impaired binding processes^18–22^. For example, research has found poor performance in the older participants in the recognition of various associated stimuli^21^. In a recent meta-analysis about aging and different memory tasks, age differences were shown to be even larger for free recall tasks compared to item recognition, suggesting a specific deficit of older adults in recalling objects from memory^19^. Thus, even after repeated practice, older users still had difficulty recalling arbitrary bindings between gestures and letters, especially under high memory load.

The present study also showed that larger memory loads were associated with lower gesture evaluation scores (i.e., easiness of learning and memorizing, likelihood to reuse, likelihood to recommend and overall gesture satisfaction), poorer emotional experience and a higher level of fatigue. However, there were no significant differences in every subjective rating between the two user groups for each memory load, despite older users actually committing significantly more errors in the recall test compared to younger users. The mismatch between gestures’ subjective evaluation ratings and actual task performance in older users has been observed in past usability studies^29,30^. For example, Giassi and Seabra^29^ reported that though the elderly over 60-years-old had more difficulty in tasks than younger adults (e.g., old users took a longer time following a new contact on social network), they evaluated the tasks as “easy” and “reproducible” as younger users. Sonderegger et al.^30^ showed similar results that older adults’ perception of a touchscreen smartphone was rather positive despite their worse performance in a typing task using the touchscreen. Thus, Sonderegger et al.^30^ speculated that when older adults could complete the task successfully (regardless of how long it takes) with such a new device, their positive experience might be associated with their increased usability ratings.

Though the sample used in the present study is sufficiently powerful to detect the effect we aimed to explore, the small sample size of both experiments might be a limitation when generalizing the results to a population with diverse characteristics and backgrounds.

## Conclusions

The present study showed that both younger and older users’ recall accuracy of the gesture-letter pairs decreased dramatically as memory load increased. In addition, memory load differentially affected the learning curve of gesture-letter pairs of younger and older users. The recall accuracy of younger users significantly improved with more sessions of memory training for all memory loads, whereas older users’ improvements in memory were not significant except the lowest memory load (i.e., 6 pairs). Furthermore, the lower memory load of gesture-letter pairs was also rated as more learnable, with better emotional experience and less fatigue as compared to the higher memory load conditions. Our work suggests an upper limit when adding new gesture-function associations on mobile phones and special attention should be devoted to old users.

## Supplementary Information

Supplementary Information.

## Data Availability

The datasets of the current study are available from the corresponding author on reasonable request.
