# Supplementary material for: Memory load differentially influences younger and older users’ learning curve of touchscreen gestures
Source: Sci Rep. 2022 Jun 25;12:10814. doi: 10.1038/s41598-022-15092-y (PMC9233678; doi:10.1038/s41598-022-15092-y)
Supplement: Supplementary file 1 — Supplementary Information. [file 41598_2022_15092_MOESM1_ESM.docx]

**Appendix A**

*Table A.* *Demographic details for samples in Experiment 1 and Experiment 2*

| Experiment | Group | *N* | Mean age/years (*SD*) | Age range/years | Gender | Touchscreen experience | Mobile OSs | Experience with fullscreen mobile phones (yes/no) |
| --- | --- | --- | --- | --- | --- | --- | --- | --- |
| **Experiment 1** | Users | 18 | 26.89 (*10.47*) | 21-38 | 8 males and 10 females | 3 had 4-6 years of experience; 15 had over 6 years of experience | 8 used iOS system and 10 used Android system | 10 "yes" and 8 "no" |
| **Experiment 2** | Younger users | 12 | 27.00 (*5.38*) | 19-38 | 5 males and 7 females | 1 had 2-4 years of experience; 4 had 4-6 years of experience; 7 had over 6 years of experience | 2 used iOS system and 10 used Android system | 11 "yes" and 1 "no" |
|  | Older users | 12 | 54.83 (*4.71*) | 45-63 | 6 males and 6 females | 1 had 1-2 years of experience; 2 had 2-4 years of experience; 2 had 4-6 years of experience; 7 had over 6 years of experience | 1 used iOS system and 11 used Android system | 7 "yes" and 5 "no" |
|  | Difference test |  | *t*(14) = -8.26, *p* < .001 |  | *χ*^2^(1, 24) = .17, *p* = .682 | *χ*^2^(3, 24) = 2.00, *p* = .572 | *χ*^2^(1, 24) = .38, *p* = .537 | *χ*^2^(1, 24) = 3.56, *p* = .059 |

**Appendix B**

**A.**

**B.**

**Figure B** Experiment 1. Gesture-letter pairs in the memory load 15 (A) and memory load 22 (B).

**Appendix C** Gesture evaluation survey

***Easiness of learning and memorizing*** *(each item is rated on a 7-point Likert scale**; 1= “very disagree”, 7= “very agree”; higher scores indicate easiness of learning and memorizing)*

- This set of gesture-letter pairs is easy to learn.
- I think I could still remember the set of gesture-letter pairs even after the test is done.
- It is difficult to learn and remember the set of gesture-letter pairs, so I need extra time to learn them well. (reversed)
- I feel confused about the set of gesture-letter pairs learned from the test. (reversed)

***Emotional experience and levels of fatigue*** *(rate between 0 and 10;* *higher scores indicate higher levels of emotional experience and fatigue)*

- The degree to which I feel happy and enjoyable during the gesture learning process.
- The degree to which I feel discouraged and annoyed during the gesture learning process.
- Please rate the levels of fatigue related to the learning process of the gestures.

***Overall evaluation*** *(rate between 0 and 10; higher scores indicate* *a higher likelihood to learn and use the set of gestures in a real interaction context, higher likelihood to recommend the gesture, and higher levels of satisfaction)*

- Please rate the likelihood to learn and use the set of gestures on touchscreen mobile phones.
- Please rate the likelihood to recommend the set of gestures to other users.
- Please rate the overall satisfaction of the gesture learning experience.

**Appendix D**

Table D. *Experiment 1. Mean (Standard Error) recall accuracy of each memory load (memory load 15, memory load 22)* *after memorizing sessions (S1, S2, S3, S4, S5).*

| Memorizing session | Memory load 15 | Memory load 22 |
| --- | --- | --- |
| S1 | 0.18 (*.05*) | 0.19 (*.02*) |
| S2 | 0.45 (*.05*) | 0.33 (*.05*) |
| S3 | 0.58 (*.06*) | 0.41 (*.06*) |
| S4 | 0.68 (*.07*) | 0.52 (*.06*) |
| S5 | 0.72 (*.07*) | 0.62 (*.07*) |
| Average | 0.52 (*.03*) | 0.41 (*.03*) |

**Appendix E**

Table E. *Experiment 1. Mean (SE) subjective rating scores in each memory load (memory load 15, memory load 22).*

| Subjective ratings |  | Memory load 15 | Memory load 22 |
| --- | --- | --- | --- |
| Gesture evaluation | *Easiness of learning and memorizing* | 2.72 (*.27*) | 2.39 (*.23*) |
|  | *Likelihood to use* | 3.56 (*.64*) | 2.22 (*.55*) |
|  | *Likelihood to recommend* | 3.00 (*.52*) | 1.39 (*.34*) |
|  | *Overall satisfaction* | 4.00 (*.67*) | 3.39 (*.60*) |
| Fatigue and emotional experience | *Positive emotion* | 3.72 (*.62*) | 2.56 (*.53*) |
|  | *Negative emotion* | 4.22 (*.76*) | 5.67 (*.68*) |
|  | *Fatigue* | 5.28 (*.71*) | 6.67 (*.66*) |

**Appendix F**

Version 1

Version 2

Version 3

Version 4

Version 5

Version 6

**Figure F**. Experiment 2. Gesture-letter pairs in the memory load 6, 9, 18 and 22. A user learned only one version of the gesture-letter association including the four memory load conditions. Versions learned were counterbalanced across users.

**Appendix G**

Table G. *Experiment 2. Mean (Standard Error) recall accuracy of each memory load (memory load 6, memory load 9, memory load 18, memory load 22) after memorizing sessions (S1, S2, S3, S4, S5) in the two user groups (younger, older).*

| Group | Memorizing session | Memory load 6 | Memory load 9 | Memory load 18 | Memory load 22 |
| --- | --- | --- | --- | --- | --- |
| Younger users | S1 | 0.65 (*.07*) | 0.44 (*.09*) | 0.26 (*.06*) | 0.16 (*.04*) |
|  | S2 | 0.81 (*.06*) | 0.69 (*.08*) | 0.41 (*.09*) | 0.28 (*.05*) |
|  | S3 | 0.89 (*.05*) | 0.83 (*.07*) | 0.57 (*.10*) | 0.44 (*.07*) |
|  | S4 | 0.92 (*.08*) | 0.89 (*.07*) | 0.63 (*.11*) | 0.53 (*.09*) |
|  | S5 | 0.85 (*.09*) | 0.96 (*.02*) | 0.63 (*.10*) | 0.60 (*.10*) |
|  | Average | 0.82 (*.03*) | 0.76 (*.04*) | 0.50 (*.05*) | 0.40 (*.04*) |
| Older users | S1 | 0.32 (*.08*) | 0.36 (*.08*) | 0.19 (*.05*) | 0.16 (*.07*) |
|  | S2 | 0.42 (*.07*) | 0.42 (*.09*) | 0.21 (*.07*) | 0.19 (*.06*) |
|  | S3 | 0.54 (*.08*) | 0.49 (*.09*) | 0.25 (*.72*) | 0.27 (*.07*) |
|  | S4 | 0.50 (*.09*) | 0.57 (*.09*) | 0.28 (*.07*) | 0.28 (*.09*) |
|  | S5 | 0.68 (*.09*) | 0.67 (*.09*) | 0.32 (*.06*) | 0.31 (*.10*) |
|  | Average | 0.49 (*.04*) | 0.50 (*.04*) | 0.25 (*.03*) | 0.24 (*.04*) |

**Appendix H**

Table H. *Experiment2. Results of four two-way ANOVAs on recall accuracy in the four memory loads, using memorizing session as within-subject factor and user group as between-subject factor.*

|  | Memory load 6 | | | | | Memory load 9 | | | |
| --- | --- | --- | --- | --- | --- | --- | --- | --- | --- |
| Factor | *F* | *df* | *p* | *η^2^_p_* | *F* | | *df* | *p* | *η^2^_p_* |
| Memorizing session | 7.08 | 4, 88 | **<.001** | .24 | 25.49 | | 4, 88 | **<.001** | .54 |
| Group | 20.38 | 1, 22 | **<.001** | .48 | 7.21 | | 1, 22 | **.014** | .25 |
| Memorizing session×Group | 1.37 | 4, 88 | .248 | .06 | 2.74 | | 4, 88 | **.034** | .11 |
|  | Memory load 18 | | | | | Memory load 22 | | | |
|  | *F* | *df* | *p* | *η^2^_p_* | *F* | | *df* | *p* | *η^2^_p_* |
| Memorizing session | 16.79 | 4, 88 | **<.001** | .43 | 23.98 | | 4, 88 | **<.001** | .52 |
| Group | 5.82 | 1, 22 | **.025** | .21 | 2.64 | | 1, 22 | .118 | .11 |
| Memorizing session×Group | 5.17 | 4, 88 | **<.001** | .19 | 5.45 | | 4, 88 | **<.001** | .20 |

A two-way ANOVA with mixed measurements was run in each of the four memory loads. The within-subject factor was memorizing session and the between-subject factor was user group. Statistical results are shown in Table H.

For the memory load 6, younger users on average (0.82) outperformed older users (0.49) in the recall accuracy. Post hoc comparisons corrected by Holm revealed that after repeated learning and memorizing users’ memory performance was significantly enhanced compared to their first recall test (S1 vs S2, *p* = .065; S1 vs S3, *p* < .001; S1 vs. S4, *p* = .022, S1 vs. S5, *p* = .007).

For the size set 9, younger users (0.76) indicated higher accuracy compared to older users (0.50). Memory performance significantly improved in younger users after repeated learning and memorizing (S1 vs S2, *p* = .015; S1 vs S3, *p* = .007; S1 vs. S4, *p* = .012, S1 vs. S5, *p* = .002), whereas older users did not show increased memory performance over memorizing sessions (for all comparisons, *p* > .05).

For the memory load 18, higher accuracy was found in younger (0.50) than in older users (0.25). With several memorizing sessions memory performance significantly improved in the younger users (S1 vs S4, *p* = .040; S1 vs S5, *p* = .029), but not in the older counterparts (for all comparisons, *p* > .05).

For the memory load 22, both groups did not differ significantly in recall accuracy. With several memorizing sessions memory performance significantly improved in the younger (S1 vs S3, *p* = .007; S1 vs S4, *p* = .004; S1 vs S5, *p* = .004), but not in the older users (for all comparisons, *p* > .05).

**Appendix I**

Table I. *Experiment 2. Mean (SE) subjective rating scores in each memory load (memory load 6, memory load9, memory load 18, memory load 22).*

| Subjective ratings |  | Memory load 6 | Memory load 9 | Memory load 18 | Memory load 22 |
| --- | --- | --- | --- | --- | --- |
| Gesture evaluation | *Easiness of learning and memorizing* | 3.32 (.31) | 3.32 (*.28*) | 2.76 (*.22*) | 2.26 (*.24*) |
|  | *Likelihood to use* | 3.58 (*.55*) | 4.29 (*.55*) | 3.00 (*.55*) | 2.29 (*.50*) |
|  | *Likelihood to recommend* | 4.13 (*.49*) | 4.29 (*.52*) | 3.17 (*.54*) | 2.29 (*.54*) |
|  | *Overall satisfaction* | 4.92 (*.62*) | 5.08 (*.55*) | 3.29 (*.49*) | 3.38 (*.61*) |
| Fatigue and emotional experience | *Positive emotion* | 4.00 (*.51*) | 4.46 (*.61*) | 2.71 (*.43*) | 2.17 (*.42*) |
|  | *Negative emotion* | 4.04 (*.53*) | 4.25 (*.61*) | 5.33 (*.59*) | 6.00 (*.52*) |
|  | *Fatigue* | 4.46 (*.56*) | 4.54 (*.58*) | 5.88 (*.58*) | 6.46 (*.52*) |
